# Equity of Access to Quality of Care in Family Medicine

**DOI:** 10.1155/2011/858131

**Published:** 2012-02-26

**Authors:** M. F. Harris, J. S. Furler, S. W. Mercer, S. J. Willems

**Affiliations:** ^1^Centre for Primary Health Care and Equity, Faculty of Medicine, UNSW, Sydney, NSW 2052, Australia; ^2^Department of General Practice, The University of Melbourne, Parkville, VIC 3052, Australia; ^3^Section of General Practice and Primary Care, University of Glasgow, Glasgow G12 8QQ, UK; ^4^Department of General Practice and Primary Health Care, Ghent University, De Pintelaan 185 1K3, 9000 Gent, Belgium

Accessibility is an important characteristic of primary health care contributing in large part to its success in making health care more efficient and equitable. Countries which have comprehensive primary health care systems have lower costs and generally healthier populations [1, 2]. To get the benefits of PHC services, people need to be able to access and use them. Access to primary health care has also been proposed as a strategy to address health inequities [3]. 

Access varies in different contexts. In some countries, there are major barriers to access to basic primary health care. In others, even though there is reasonably equitable access *to* primary health care, there are inequitable barriers to accessing quality or comprehensive care and to subsequent referral services, sometimes referred to as access *in* care. “Inverse Care” is where persistent barriers to access exist for people from disadvantaged backgrounds, despite their higher need [4]. Where such inverse care persists, health care can inadvertently become part of the system that sustains unjust inequities in health-making action at this level imperative [5].

Access arises from a complex interplay between patient, provider, and system factors. Accessibility can be defined as the opportunity or ease with which consumers or communities are able to use services in proportion to their need [6]. Patient factors include economic resources, health literacy, and attitudes. Provider and system factors are closely linked and have been summarised by 5As originally described by Pechansky and others [7, 8] as follows.

**Availability**

of a sufficient volume of services (including professionals, facilities, and programmes) to match the needs of the population and the location of services close to those needing them. This is underpinned by the distribution of the health workforce.

**Affordability**

(cost versus consumers' ability to pay, impact of health care costs on socioeconomic circumstances of patients). This is influenced by the way in which government funds primary health care and the regulation of and access to the health insurance.

**Accommodation**

the delivery of services in such a manner that those in need of them can use them without difficulty (e.g., appropriate hours of opening and accessible buildings).

**Appropriateness**

to socioeconomic, educational, cultural, and linguistic needs of patients.

**Acceptability**

in terms of consumer attitudes and demands.

This special issue presents a number of studies of the complex interplay of all these patient, provider, and system factors. Two papers deal with access to mental health services. In a qualitative study in northwestern England, K. Bristow et al. in “*Help seeking and access to primary care for people from “hard-to-reach” groups with common mental health problems*” identify four factors which influence access to mental health care by “hard-to-reach groups” including patient conceptualisation of health care and their help seeking behaviours as well as barriers such as lack of time and the challenge of negotiating a range of services. Patients hoped for a GP willing to listen and refer or liaise with specialist services but did not always get this due to lack of GP time and linkages with social care and nongovernment organizations, something that other research has demonstrated to be a barrier in disadvantaged communities, and it has been suggested that colocation of services may be a useful strategy to address this [9]. The second paper J. Benson et al. “*A new era in mental health care in Vanuatu*” describes access to mental healthcare in a small Pacific Island country with a less developed economy—Vanuatu. In this setting, traditional models of specialised mental health services are unsustainable. The paper describes an approach to training key health and social service providers and the provision of a support network for them. 

Another group who suffer access problems in many countries are minority ethnic population groups. In a mixed methods study in the Netherlands, C. H. Liu et al. in “*Barriers to health care for Chinese in the Netherlands*” describe access barriers for people of Chinese background to all primary health care including mental health problems. Important barriers include inadequate knowledge of the health system that leads to nonregistration with GPs and language barriers (with inadequate access to interpreters).

The need for workforce development has given rise to a number of innovative solutions which involve other kinds of health professionals. In North America, physician assistants have been developed to address the shortage of doctors in health services in many areas. However, like the medical workforce, the distribution of physician assistants is also subject to inequities. This is addressed in the paper by J. M. Coombs et al. “*Factors associated with physician assistant practice in rural and primary care in Utah*” in a survey of physician assistants in Utah. While making a significant contribution to the primary care workforce, deficiencies in rural areas remain. They noted that PAs who grew up in rural areas were more likely to practice in rural areas. This has important implications for recruitment and retention strategies and supports extension of some of the strategies used to attract and retain the rural medical workforce.

Funding is another key determinant of availability and affordability. C. Teljeur et al. in “*Spatial variation in general medical services income in Dublin general practitioners*” have examined the allocation of government funding for general practice services in Dublin. Although, as a whole, GPs practicing in disadvantaged areas attract more total funding, the provision of universal funding for care for patients over 70 years of age tends to be skewed towards more advantaged areas. This raises not only issues about implications of this in support for primary health care in disadvantaged areas but also optimal mix of universal and targeted funding for health care more generally and the impact this can have on the distribution of the workforce. Almost all countries have problems regarding the distribution of the workforce in disadvantaged compared with advantaged communities, and the mechanism for funding for primary health care has an important influence on the choices doctors make about where to practice.

Finally, V. Bercic et al. in “*Development of a tool to identify poverty in a family practice setting: a pilot study*” in Canada developed a simple set of acceptable questions that clinicians can ask to determine if patients are affected poverty. This allows the detection of important causes of inequities and potentially enables affordability issues to be more directly discussed in the consultation—especially affordability of referral services or treatments. This emphasizes the importance of not only describing and advocating inequities through professional bodies but also of clinicians playing a direct role in addressing inequities of access affecting their own patients. 

The British Medical Association recently published a paper describing the role of the doctor to address the social determinants of health [9]. In his forward, Michael Marmot stresses that addressing access is only part of the picture and that health inequalities are related to a range of structural determinants such as age, income, education, occupation, gender, ethnicity, and place of residence. However, he gives examples of measures to improve access for groups such as homeless people and access to interpreter services for people facing language barriers to care. While not the whole solution, retaining and improving equitable access to primary health remains an important priority for health systems and one which should not be taken for granted. 

*M. F. Harris* *M. F. Harris* *J. S. Furler* *J. S. Furler* *S. W. Mercer* *S. W. Mercer* *S. J. Willems* *S. J. Willems*
